# Regularized Gradient Statistics Improve Generative Deep Learning Models of Super Resolution Microscopy

**DOI:** 10.1002/smtd.202401900

**Published:** 2025-06-02

**Authors:** Meri Abgaryan, Xinning Cui, Nandu Gopan, Gabriel della Maggiora, Artur Yakimovich, Ivo F. Sbalzarini

**Affiliations:** ^1^ Dresden University of Technology Faculty of Computer Science 01187 Dresden Germany; ^2^ Max Planck Institute of Molecular Cell Biology and Genetics 01307 Dresden Germany; ^3^ Center for Systems Biology Dresden 01307 Dresden Germany; ^4^ Center for Advanced Systems Understanding (CASUS) 02826 Görlitz Germany; ^5^ Helmholtz‐Zentrum Dresden‐Rossendorf (HZDR) 01328 Dresden Germany; ^6^ Institute of Computer Science University of Wrocław Wrocław 50‐300 Poland; ^7^ Cluster of Excellence “Physics of Life” TU Dresden 01062 Dresden Germany; ^8^ Center for Scalable Data Analytics and Artificial Intelligence, ScaDS.AI Dresden/Leipzig 01062 Dresden Germany; ^9^ School of Computation Information and Technology Technical University of Munich 85748 Munich Germany

**Keywords:** deep learning, diffusion models, generative artificial intelligence, image quality, super‐resolution microscopy

## Abstract

It is shown that regularizing the signal gradient statistics during training of deep‐learning models of super‐resolution fluorescence microscopy improves the generated images. Specifically, regularizing the images in the training data set is proposed to have gradient and Laplacian statistics closer to those expected for natural‐scene images. The BioSR data set of matched pairs of diffraction‐limited and super‐resolution images is used to evaluate the proposed regularization in a state‐of‐the‐art generative deep‐learning model of super‐resolution microscopy, the Conditional Variational Diffusion Model (CVDM). Since the proposed regularization is applied as a preprocessing step to the training data, it can be used in conjunction with any supervised machine‐learning model. However, its utility is limited to images for which the prior is appropriate, which in the BioSR data set are the images of filamentous structures. The quality and generalization power of CVDM trained with and without the proposed regularization are compared, showing that the new prior yields images with clearer visual detail and better small‐scale structure.

## Graphical abstract

A generative AI model for super‐resolution microscopy images is presented. Super‐resolution microscopy provides high spatial detail at the expense of lower time resolution. Using it for live samples requires computational image reconstruction. It is unclear what good priors and metrics for AI‐generated super‐resolution images are. Here, a perceptive metric accounting for the human visual system provides clues.

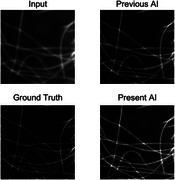

## 1 Introduction

Super‐resolution microscopy (SRM)^[^ ^1^, ^2^, ^3^, ^4^ ^]^ encompasses a set of advanced fluorescence microscopy techniques capable of resolving structural details below the diffraction limit of conventional light microscopy. These methods achieve enhanced spatial resolution by acquiring multiple images of the same sample and reconstructing a super‐resolved image. This comes at the expense of lower temporal resolution or smaller field of view, limiting the applicability of SRM in live samples.

Super‐resolution imaging of live samples requires computational SRM approaches.^[^ ^2^, ^4^ ^]^ Traditionally, this was formulated as a deconvolution problem,^[^ ^5^ ^]^ wherein diffraction blur is removed by deconvolving the image with the inverse point‐spread function of the microscope.^[^ ^6^ ^]^ Deconvolution is an ill‐posed problem, as the inverse point‐spread function is inherently unstable, and the point‐spread function is not only determined by the microscope optics but also by the unknown and spatially varying optical properties of the sample. Thus, reconstructing a super‐resolution image from a diffraction‐limited input constitutes an inverse problem requiring additional regularization. Such regularization is typically incorporated as prior assumptions about the ground truth.

The prior assumptions about the desired output image should neither be too strong nor too weak. When computationally reconstructing a SRM image from a diffraction‐limited image, geometric priors about the shapes or appearance of the imaged objects are typically too strong, leading to visible bias artifacts and to a lack of image detail. General deep‐learning priors, such as cross entropy or total variation, tend to be too weak and can lead to unstable reconstructions or amplification of imaging noise. As a middle ground, spectral priors have been proposed to provide the right amount of regularization for light‐microscopy images.^[^ ^7^ ^]^ A spectral prior makes no assumptions about the specific appearance of image contents, but rather about the statistical distribution of image intensity gradients or higher derivatives.

Regularizing image intensity distributions using a spectral prior requires specifying a target distribution toward which desirable output images shall be biased. This target distribution must be known a priori and be independent of the input and training data. If the images are intended for visual inspection, to facilitate discovery by humans, image‐reconstruction priors ideally also account for the capability of the human visual system. Like any signal‐receiving system, the human visual system has evolutionarily adapted to signals of a certain input gradient statistic,^[^ ^8^, ^9^ ^]^ embodying a spectral prior. Indeed, human vision relies on gradient information for key perceptual tasks such as edge detection, contour recognition, and texture perception.^[^ ^10^, ^11^, ^12^, ^13^ ^]^ The statistical properties of gradient distributions in natural‐scene images have been evolutionarily encoded in the receptive fields of cortical neurons.^[^ ^14^, ^15^ ^]^ These distributions universally follow a hyper‐Laplace law,^[^ ^8^, ^15^ ^]^ which can be used as a spectral prior in image‐generation models. This has previously proven beneficial in light‐microscopy^[^ ^7^ ^]^ and general image‐reconstruction tasks.^[^ ^16^ ^]^ A spectral prior that biases the intensity gradient distribution toward the one expected for natural‐scene images is called a naturalness prior.^[^ ^17^ ^]^

Here, we propose using a naturalness prior when training generative deep‐learning models of SRM. Naturalness priors are well suited for deep learning, since they are independent of the training and input images and can be evaluated for any given candidate image without requiring a reference image. Deep learning has demonstrated significant potential in light‐microscopy imaging,^[^ ^18^, ^19^, ^20^, ^21^ ^]^ and Deep‐Learning Super‐Resolution (DLSR) methods are among the most promising approaches to computational SRM. These methods infer a super‐resolution reconstruction from a diffraction‐limited input image using methods from generative machine learning, such as Generative Adversarial Networks (GANs),^[^ ^22^ ^]^ regression networks,^[^ ^23^ ^]^ diffusion models,^[^ ^24^, ^25^ ^]^ and generative variational autoencoders.^[^ ^26^ ^]^ This provides a family of DLSR methods using different neural network architectures. Each of these DLSR architectures comes with its own set of advantages and limitations: Regression‐based methods often suffer from reconstruction loss,^[^ ^27^ ^]^ noise, and overfitting.^[^ ^28^ ^]^ GAN‐based methods, while capable of generating high‐fidelity images, are prone to hallucination artifacts, such as unnatural textures,^[^ ^29^ ^]^ and mode collapse.^[^ ^30^ ^]^ Classic diffusion models better approximate the target distribution, but their performance depends on manually tuned variance schedules. More recent diffusion models, such as consistency trajectory models^[^ ^31^ ^]^ and neural flow diffusion models^[^ ^32^ ^]^ address these limitations. Variational Diffusion Models,^[^ ^33^, ^34^ ^]^ including Conditional Variational Diffusion Models (CVDM),^[^ ^24^ ^]^ learn both the variance schedule and the conditional probability distribution from data. This makes CVDM particularly promising for DLSR applications.^[^ ^24^ ^]^

**Figure 1:**
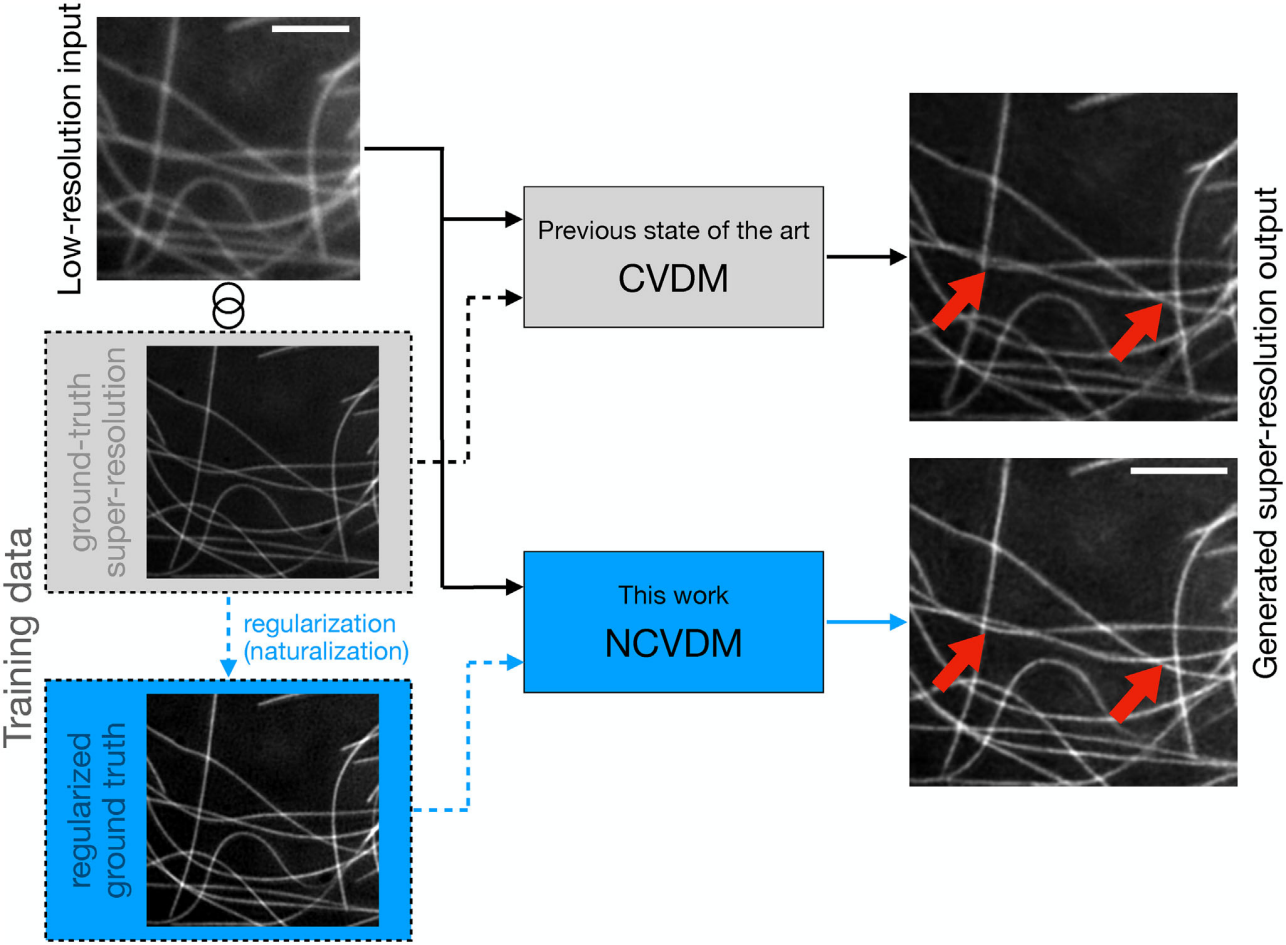
Generative deep‐learning models, such as Conditional Variational Diffusion Models (CVDM), can predict super‐resolution fluorescence microscopy images (right column) from diffraction‐limited low‐resolution input images (top left).^[^ ^24^ ^]^ For this, the machine‐learning model is first trained on matched pairs of low‐resolution and super‐resolution images of the same structures (“Training data”, dashed lines). Here, we propose regularizing the training images (blue dashed lines). Specifically, we regularize the intensity gradient statistics of the training images to match those expected for natural‐scene images using a data preprocessing step called “naturalization”. The resulting generative model—naturalized CVDM (NCVDM)—outputs super‐resolution images with better texture and detail in diffraction‐limited regions (red arrowheads). Arrows show data flow for inference (solid lines) and training (dashed lines); gray denotes the previous state of the art; blue highlights the present contribution. The example image from the BioSR dataset^[^ ^22^ ^]^ shows microtubules. The scale bars are 1.5 µm.

We investigate the utility of a naturalness prior in CVDM‐based DLSR models as illustrated in **Figure** **1**. Specifically, we show that training CVDM on images whose gradient statistics better match those of natural‐scene images improves the DLSR model's ability to reconstruct small‐scale image details. We hypothesize that this is due to lower spectral bias, a phenomenon by which deep‐learning models tend to neglect high‐frequency signal components.^[^ ^35^, ^36^ ^]^ In images, high‐frequency signal components correspond to small‐scale structures. Accurately reconstructing small‐scale structures, such as those highlighted by the red arrowheads in Figure 1 on the right, is the primary purpose of SRM. This is why spectral bias has been reported as a major concern in DLSR models.^[^ ^24^ ^]^ We believe that DLSR models are less prone to spectral bias when trained on images with more natural gradient statistics.

We impose natural gradient statistics as a spectral prior to regularize the training data prior to model training, as shown in Figure 1 on the left. Alternatively, priors can be included in the loss functional of the machine‐learning model. This, however, limits their applicability to certain types of models. In particular, it is not straightforward to include a spectral prior in the loss of a generative diffusion model, such as CVDM. We instead train a CVDM on images with more natural gradient statistics by “naturalizing” the training images in a preprocessing step. Image naturalization uses gradient‐histogram equalization to transform an image into a more “natural” one, i.e., one that has gradient statistics closer to natural scene images.^[^ ^16^ ^]^ Using naturalized images for CVDM training results in “naturalized CVDM” (NCVDM, blue in Figure 1). Since the parametric form of natural‐scene gradient distributions is known to be a hyper‐Laplacian,^[^ ^8^, ^15^ ^]^ the distance between the gradient distribution of a given image and the target naturalness prior can be expressed by a single scalar number, called the naturalness factor, *N*_*f*_.^[^ ^16^ ^]^

In order to use the naturalness factor as a regularizer in DLSR using CVDM, we address three points: First, we show that the naturalness factor is not redundant with classic image quality metrics. Specifically, we show that the *N*_*f*_ of an image cannot reliably be predicted from the mean absolute error (MAE),^[^ ^37^ ^]^ peak signal‐to‐noise ratio (PSNR),^[^ ^38^ ^]^ multi‐scale structural similarity index measure (MS‐SSIM),^[^ ^39^ ^]^ root mean square contrast,^[^ ^40^ ^]^ or Fourier ring correlation resolution.^[^ ^41^, ^42^ ^]^ Second, we show that *N*_*f*_ reflects the perceived quality of CVDM‐generated DLSR images of sub‐diffraction structures for which the ground‐truth high‐resolution images are known to have high naturalness. Third, we show that including *N*_*f*_ = 1 as a prior into CVDM training improves image detail reconstruction and texture.

These results suggest that using a naturalness prior to regularize gradient statistics when training DLSR models can improve the quality and interpretability of the generated images.

## 2 Experimental Section

The naturalness factor was proposed as a practically computable prior for DLSR model training. It was computed over the cumulative gradient distribution (CGD) and the cumulative Laplacian distribution (CLD) of all pixels in an image. Both distributions were approximated by their normalized discrete histograms over pixel‐wise image intensity gradients and Laplacians computed using compact finite differences on the pixel grid. In total, 23,613 eight‐bit natural scene images were used for estimating the target distribution,^[^ ^16^ ^]^ which we re‐used. In order to re‐use the published prior, images were converted to eight‐bit when determining *N*_*f*_, while all other processing and the deep‐learning model itself use 32‐bit precision. It had been shown that the CGD and CLD follow hyper‐Laplace probability distributions parameterized by a scalar *T*.^[^ ^7^ ^]^ The natural‐scene parameter for the CGD is $T_{1}^{\text{pr}}$= 0.38 and for the Laplacian $T_{2}^{\text{pr}}$= 0.14, as computed by **Algorithm** **1**. For each image to test, *T*_1_ and *T*_2_, were computed using **Algorithm** **2**, and the naturalness factor *N*_*f*_ of the image is defined as:^[^ ^16^ ^]^

(1) $$N_{f}=\left(1-\theta \right)\frac{T_{1}}{T_{1}^{\text{pr}}}+\theta \frac{T_{2}}{T_{2}^{\text{pr}}}\;$$

here, θ represents the weight between the first and second‐order distributions, with θ ∈ [0, 1]. Throughout this paper, θ = 0.5 is used in accordance with previous works.^[^ ^7^, ^16^ ^]^

The scalar *N*_*f*_ quantifies the similarity between the gradient and Laplacian distributions of an image and the respective prior distributions for natural‐scene images. Values close to one correspond to image with more natural‐scene‐like distributions. Images with *N*_*f*_ > 1 have more small gradients than natural‐scene images, images with *N*_*f*_ < 1 more large gradients. A key advantage of this metric is that, once the priors were found, it did not require a ground truth image in order to be computed.

Algorithm 1 **Naturalness Prior Algorithm.**

**Table:** 

**Require**: natural image data set, image count *n*	
1:	**for** each image *I*_*i*_(*x*, *y*) **do**
2:	convert image to 8‐bit gray‐scale
3:	calculate gradient field: $G_{x}^{i}(x,y)=I_{i}\left(x+1,y\right)-I_{i}\left(x,y\right)$, $G_{y}^{i}(x,y)=I_{i}\left(x,y+1\right)-I_{i}\left(x,y\right)$
4:	calculate marginal distribution: $G_{i}^{x}$, $G_{i}^{y}$
5:	calculate Laplace field: *L*_*i*_(*x*, *y*) = Δ*I*_*i*_(*x*, *y*)
6:	calculate $T_{1}^{i}$ by fitting the CGD: $\tilde{C}\left(G\right)=\left(\frac{1}{2}+\frac{\mathrm{atan}(T_{1}G^{x})}{\pi }\right)\left(\frac{1}{2}+\frac{\mathrm{atan}(T_{1}G^{y})}{\pi }\right)$
7:	calculate $T_{2}^{i}$ by fitting the CLD: $\tilde{L}\left(t\right)=\frac{\mathrm{atan}(T_{2}t)}{\pi }+\frac{1}{2}$
8:	**end for**
9:	compute the average prior: $T_{1}^{\text{pr}}=\frac{1}{n}\sum_{i=1}^{n}T_{1}^{i}$ and $T_{2}^{\text{pr}}=\frac{1}{n}\sum_{i=1}^{n}T_{2}^{i}$

Algorithm 2 **Naturalness Factor Algorithm.**

**Table:** 

**Require**: images, $T_{1}^{\text{pr}}=0.38$, $T_{2}^{\text{pr}}=0.14$, weight parameter θ ∈ [0, 1]	
1:	**for** each image *I*_*i*_(*x*, *y*) **do**
2:	convert image to 8‐bit gray‐scale
3:	calculate $T_{1}^{i}$ and $T_{2}^{i}$ as in Algorithm 1
4:	calculate $N_{f}^{i}=\left(1-\theta \right)\frac{T_{1}^{i}}{T_{1}^{\text{pr}}}+\theta \frac{T_{2}^{i}}{T_{2}^{\text{pr}}}$.
5:	**end for**

To evaluate the utility of the naturalness factor in DLSR, the publicly available BioSR data set were used.^[^ ^22^ ^]^ The BioSR biological image data set for SRM contains over 2,200 pairs of matching low‐resolution and super‐resolution images across four biological structures (clathrin‐coated pits (CCP), endoplasmic reticulum (ER), microtubules (MT), and F‐actin filaments), nine signal levels (15…600 average photon count), and two SRM methods (linear and nonlinear Structured Illumination Microscopy (SIM)), the latter only for F‐actin. For every condition, a set of 50 SIM images was provided. Each of these sets of SIM images was averaged to a diffraction‐limited wide‐field image, from which the ground‐truth SIM image was to be reconstructed.

As a generative model for DLSR reconstruction, the state‐of‐the‐art CVDM was used,^[^ ^24^ ^]^ which was re‐trained on the BioSR data set. For this, the BioSR data set is partitioned into disjoint training and test sets of image pairs. After standard data augmentation and cropping to a uniform image size of 256×256 pixel, this results in 101,880 pairs of low‐resolution and super‐resolution images that were used for training the deep‐learning models. Further details about the models and their training were given in the Appendix. The same hyperparameters and training schedule was used for all models compared in this paper.

The quality of the images generated by the trained models was evaluated using the test data set, consisting of 8,544 image pairs (2,136 for each of the four biological structures). For testing, the respective low‐resolution image was input to the trained model, and the model output was compared with the corresponding ground‐truth super‐resolution image using several image‐quality metrics. This included the mean absolute error (MAE),^[^ ^37^ ^]^ which took two images as input and calculates the averaged absolute pixel‐wise difference between them. In this case, the normalized generated image $\hat{y}$ and the corresponding normalized ground truth *y* are compared as:

(2) $$\text{MAE}\left(\hat{y},y\right)=\frac{1}{n}\sum_{i=1}^{n}\left|y_{i}-{\hat{y}}_{i}\right|$$

over all pixels *i* = 1, …, *n* of the images. Lower MAE values indicate images closer to ground truth, with MAE = 0 corresponding to identical images.

The second evaluation metric was the multi‐scale structural similarity index measure, MS‐SSIM.^[^ ^39^ ^]^ It assessed the structural similarity of two images across multiple scales. Images went through a low‐pass filter and get iteratively downsampled by a factor of two. The original images were indexed with scale 1. The images obtained by downsampling were indexed with scales 2, …, *M* for a total of *M* − 1 downsampling steps. At every scale, the standard SSIM^[^ ^43^ ^]^ was calculated. MS‐SSIM was obtained by calculating a weighted average SSIM across scales:

(3) $$\text{MS-SSIM}\left(\hat{y},y\right)={\left[l_{M}\left(\hat{y},y\right)\right]}^{\alpha_{M}}\cdot \prod_{j=1}^{M}{\left[c_{j}\left(\hat{y},y\right)\right]}^{\beta_{j}}{\left[s_{j}\left(\hat{y},y\right)\right]}^{\gamma_{j}}$$

where *l*_*M*_, *c*_*j*_, and *s*_*j*_ are luminance, contrast, and SSIM at scale *j*, respectively and α_*j*_ = β_*j*_ = γ_*j*_ = {0.0448, 0.2856, 0.3001, 0.2363, 0.1333} for the scales *j* = 1, …, 5.^[^ ^39^ ^]^ In addition, the cross‐scale weights were normalized such that $\sum_{j=1}^{M}\gamma_{j}=1$. MS‐SSIM was computed using the implementation available in TensorFlow.^[^ ^44^ ^]^ The MS‐SSIM ranges between 0 and 1 with higher values indicating more similar images.

The third metric used was the peak signal‐to‐noise ratio (PSNR).^[^ ^38^ ^]^ It quantified the ratio between the maximum possible power of a signal and the power of corrupting noise on a logarithmic scale:

(4) $$\text{PSNR}=10\cdot \log_{10}\left(\frac{{\text{MAX}}_{I}^{2}}{\text{MSE}}\right)$$

where MAX_*I*_ is the maximum possible pixel intensity in the image (e.g., 65,535 for 16‐bit images) and MSE is the mean squared error between an image $\hat{y}$ and the reference image *y*:

(5) $$\text{MSE}=\frac{1}{n}\sum_{i=1}^{n}{\left(y_{i}-{\hat{y}}_{i}\right)}^{2}\;$$

PSNR values were real numbers with higher values indicating better images. PSNR = 0 corresponds to an image in which the noise was as strong as the signal.

As a fourth metric, the root mean square (RMS) contrast was considered. It measured the standard deviation of the pixel intensities, providing a global, but structure‐insensitive measure of image contrast:^[^ ^40^ ^]^

(6) $$\text{Contrast}=\sqrt{\frac{1}{n}\sum_{i=1}^{n}{\left({\hat{y}}_{i}-\mu \right)}^{2}}\;$$

with µ the mean pixel intensity over the image. The Contrast metric could be computed without requiring a ground‐truth reference image *y*. Contrast is ⩾0 with 0 corresponding to an image where all pixels have the same intensity, and higher Contrast indicating better images.

Finally, as a fifth metric, comparison was made using an image‐resolution metric. Decorrelation analysis^[^ ^41^ ^]^ was a method for estimating image resolution that did not require a‐priori knowledge. The idea was that by normalizing the Fourier transform of the input image, the noise and signal contributions were balanced, and the information about the structure of the image was preserved in the phase. A circular binary mask was then applied in the Fourier domain in order to extract the correlation related to the original ratio of the signal and noise. The algorithm consists of two steps: In the first step, a standard edge apodization was applied to suppress high‐frequency artifacts, and the normalized Fourier transform of the image was computed. Then, the input image and the normalized image were cross‐correlated in Fourier space with Pearson correlation. The result was a scalar value between 0 and 1. In the second step of the algorithm, this operation was repeated but this time the normalized Fourier transform was filtered by a circular binary mask *M*(**k**, *r*) with radius *r* ∈ (0, 1] expressed in normalized frequencies. From this, the decorrelation function was computed:^[^ ^45^ ^]^

(7) $$d\left(r\right)=\frac{\int \int ℜ\;\left\{|I\left(k\right)|M\left(k;r\right)\right\}\;dk_{x}dk_{y}}{\sqrt{{\int \;\int |I\left(k\right)|}^{2}\;dk_{x}dk_{y}\;\int \int {\left|I_{n}\left(k\right)M\left(k;r\right)\right|}^{2}\;dk_{x}dk_{y}}}\;$$

here, **k** = [*k*_*x*_, *k*_*y*_] are the Fourier space coordinates, *I*(**k**) is the Fourier transform of the input image, and *I*_*n*_(**k**) is the normalized Fourier transform. To compute the resolution metric, a sequence of masks *M*(**k**, *r*) is applied for 50 uniformly spaced radii between (0,1]. For each filtered image, the decorrelation function *d*(*r*) is computed, and *r*_*c*_ is determined as the *r* of the maximum in *d*(*r*). The resolution estimate is then defined as:

(8) $$\text{Resolution}=\frac{2\cdot \text{pixel-size}}{r_{c}}\;$$

These calculations were performed in units of nanometers with a pixel‐size of 32.3 nm as given by the BioSR data set^[^ ^22^ ^]^ using the implementation of decorrelation analysis available in PyRes.^[^ ^42^ ^]^ The Resolution metric could be computed without knowing a ground‐truth reference image *y*. Lower values of Resolution correspond to better images with smaller length scales resolved.

## 3 Results

We first establish the baseline for our comparison by characterizing the output of standard CVDM for DLSR using the metrics above. Then, we consider the three questions: 1) whether the naturalness factor *N*_*f*_ captures redundant information with the other image metrics; 2) whether *N*_*f*_ correlates with the visual quality of CVDM‐generated DLSR images; 3) what benefits it brings to directly incorporate *N*_*f*_ = 1 as a prior during CVDM training.

### 3.1 CVDM Generates Super‐Resolution Images Close to Ground Truth

We first test whether the baseline CVDM produces an image close to the ground truth in the proposed *N*_*f*_ metric. We also compare our implementation and training of CVDM with previously published results^[^ ^24^ ^]^ using the MAE and MS‐SSIM metrics in order to validate our training and use of CVDM. The results in Appendix **Table** **A1** compare the present model with the published benchmark for all four biological structures in the BioSR data set. All metrics are comparable between our CVDM implementation and the published benchmark, indicating that our implementation works correctly and has been properly trained. Slight deviations are expected due to the stochastic nature of the diffusion process.

**Table 1:** Image quality metrics of the CVDM‐generated image from Figure 4 and of its artificially blurred version. MS‐SSIM, MAE, and PSNR are computed with respect to the ground‐truth super‐resolution image. *N*_*f*_, calculated image Resolution, and Contrast do not require a reference image and are computed image‐wise. Arrows next to metric names indicate whether higher is better (↑) or lower is better (↓). Numbers in bold indicate the best performance in that respective metric.

Image	MS‐SSIM ↑	MAE ↓	PSNR ↑	Contrast ↑	Resolution ↓	$N_{f}$
CVDM	0.746	28.975	17.562	20.156	**109.152**	**0.958**
CVDM blurred	**0.787**	**20.292**	**20.211**	**22.598**	131.892	1.636

**Table 2:** Image quality metrics for the filamentous structures in the BioSR data set across the three tested DLSR models: standard CVDM, CVDM with post‐hoc output naturalization (CVDM+N), and naturalized CVDM (NCVDM). For CVDM and CVDM+N, the metrics are computed with respect to the ground‐truth super‐resolution images. For NCVDM, the reference image is the naturalized super‐resolution ground truth. Results are reported as mean ± standard deviation over 25 images of each biological structure. Arrows next to metric names indicate whether higher is better (↑) or lower is better (↓). Numbers in bold indicate the best‐performing model in that respective metric.

**Metric**	**Structures**	**CVDM**	**CVDM+N**	**NCVDM**
**MS‐SSIM ↑**	**F‐actin**	**0.819** ± 0.053	0.806 ± 0.054	0.765 ± 0.066
	**MT**	**0.830** ± 0.088	0.812 ± 0.088	0.750 ± 0.118
**MAE ↓**	**F‐actin**	**12.402** ± 5.057	13.607 ± 4.189	18.638 ± 5.597
	**MT**	**12.148** ± 4.038	15.056 ± 3.366	20.370 ± 6.656
**PSNR ↑**	**F‐actin**	**24.229** ± 2.764	22.998 ± 2.051	20.690 ± 1.881
	**MT**	**23.692** ± 2.638	21.198 ± 1.676	19.491 ± 2.071
**Contrast ↑**	**F‐actin**	26.898 ± 5.085	31.652 ± 8.035	**32.386** ± 7.460
	**MT**	31.812 ± 4.441	**43.739** ± 6.652	39.976 ± 5.859
**Resolution ↓**	**F‐actin**	107.279 ± 2.333	106.588 ± 2.886	**106.211** ± 9.484
	**MT**	108.289 ± 1.928	**105.860** ± 2.592	112.027 ± 3.356
$N_{f}$	**F‐actin**	1.157 ± 0.425	1.028 ± 0.082	**1.012** ± 0.087
	**MT**	1.452 ± 0.394	**1.059** ± 0.130	1.076 ± 0.092

**Table A1:** Comparison of our implementation of CVDM with previously published results using the MS‐SSIM (higher is better, ↑) metric and the MAE (lower is better, ↓). Our results are reported as mean ± standard deviation over 2,136 test images for each biological structure. Better values are in bold.

**Metric**	**Structure**	**Our CVDM**	**Published CVDM** ^[^ ^24^ ^]^
	**CCP**	0.898 ± 0.076	**0.955**
**MS‐SSIM** ↑	**ER**	0.899 ± 0.131	**0.934**
	**F‐actin**	0.827 ± 0.066	**0.863**
	**MT**	0.867 ± 0.069	**0.887**
	**CCP**	0.019 ± 0.008	**0.007**
**MAE** ↓	**ER**	0.039 ± 0.031	**0.032**
	**F‐actin**	0.047 ± 0.017	**0.043**
	**MT**	0.041 ± 0.014	**0.040**

**Figure 2:**
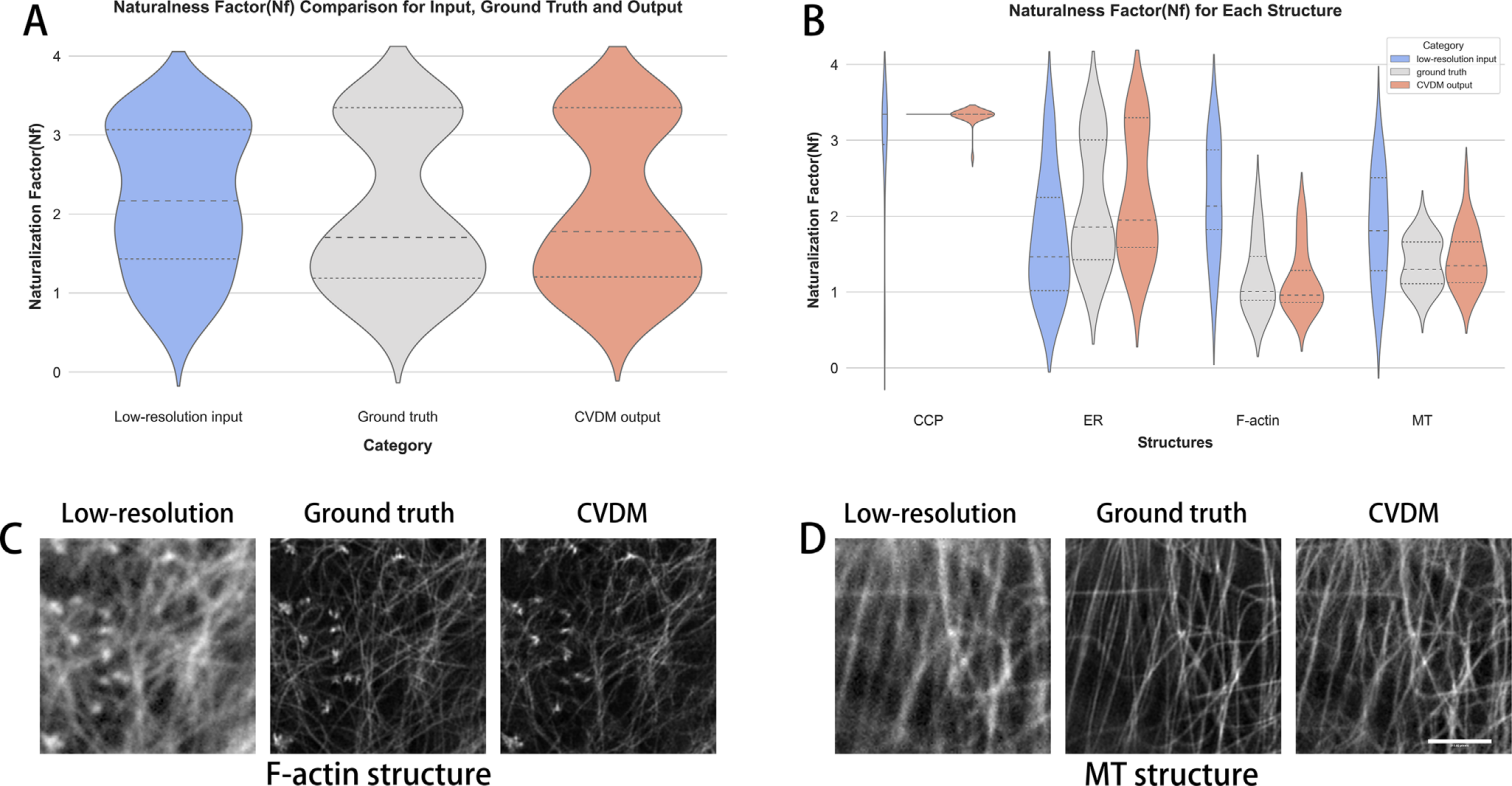
Empirical naturalness factor distributions for the different image types (low‐resolution, ground‐truth super‐resolution, CVDM‐generated) and biological structures (clathrin‐coated pits (CCP), endoplasmic reticulum (ER), F‐actin, microtubules (MT)) from the BioSR data set. A) Violin plots across 100 randomly selected images from the data set, with dashed horizontal lines indicating the median and dotted lines the quartiles of the data. B) Violin plots for the four biological structures separately with 25 randomly selected images per structure. C) Example images of F‐actin low‐resolution input, ground truth, and CVDM output (from left to right). D) Example images of microtubules (MT) low‐resolution input, ground truth, and CVDM output (from left to right). The scale bar is 1.5 µm.

We next calculate the *N*_*f*_ values for images from the BioSR data set. For this, we randomly selected 100 images from the test data set, 25 images from each of the four biological structures. We plot the *N*_*f*_ distribution for the low‐resolution images (the input), the super‐resolution images (the ground truth), and the CVDM‐generated DLSR images (the model output) in **Figure** **2**A. The ground‐truth and CVDM‐generated images show similar *N*_*f*_ distributions, while that of the input images is qualitatively different. This indicates that using *N*_*f*_ as a prior in CVDM‐based DLSR should not steer the model output away from ground truth.

We next ask the question for which biological structure a model prior of *N*_*f*_ = 1 is appropriate in generative DLSR. For this, we plot the *N*_*f*_ distributions of all three image types separately for each of the four biological structures in Figure 2B. In all cases, the distributions for the low‐resolution images are qualitatively different from those of the super‐resolution and CVDM‐generated images, confirming the finding on the whole data set. In all cases, the distributions for CVDM‐generated and ground‐truth images are again similar to each other. For CCP and ER, however, the super‐resolution images have ground‐truth *N*_*f*_ away from 1, indicating that they do not fulfill the naturalness prior. For filamentous structures (F‐actin and MT), the *N*_*f*_ distributions between super‐resolution and CVDM‐generated images differ slightly but have medians close to one. For these images of filamentous structures, imposing *N*_*f*_ = 1 as a prior in a DLSR model therefore seems appropriate. This is because they have ground‐truth *N*_*f*_ centered around one, yet the distributions differ between ground‐truth and CVDM output, indicating that a further prior could be useful.

Visual examples of images of filamentous structures are shown in Figure 2C,D. Both F‐actin and MT structures are thin, highly detailed, and often cross. The images also support the conclusion from the plots that CVDM‐generated images resemble the ground truth more closely than the low‐resolution images.

### 3.2 The Naturalness Factor is Not Redundant with Other Image Metrics

Having established the CVDM baseline, we test our first claim, namely that *N*_*f*_ quantifies information that is not redundant with one of the other metrics. Of particular interest is the relation between *N*_*f*_ and Contrast, as well as calculated image Resolution.

**Figure 3:**
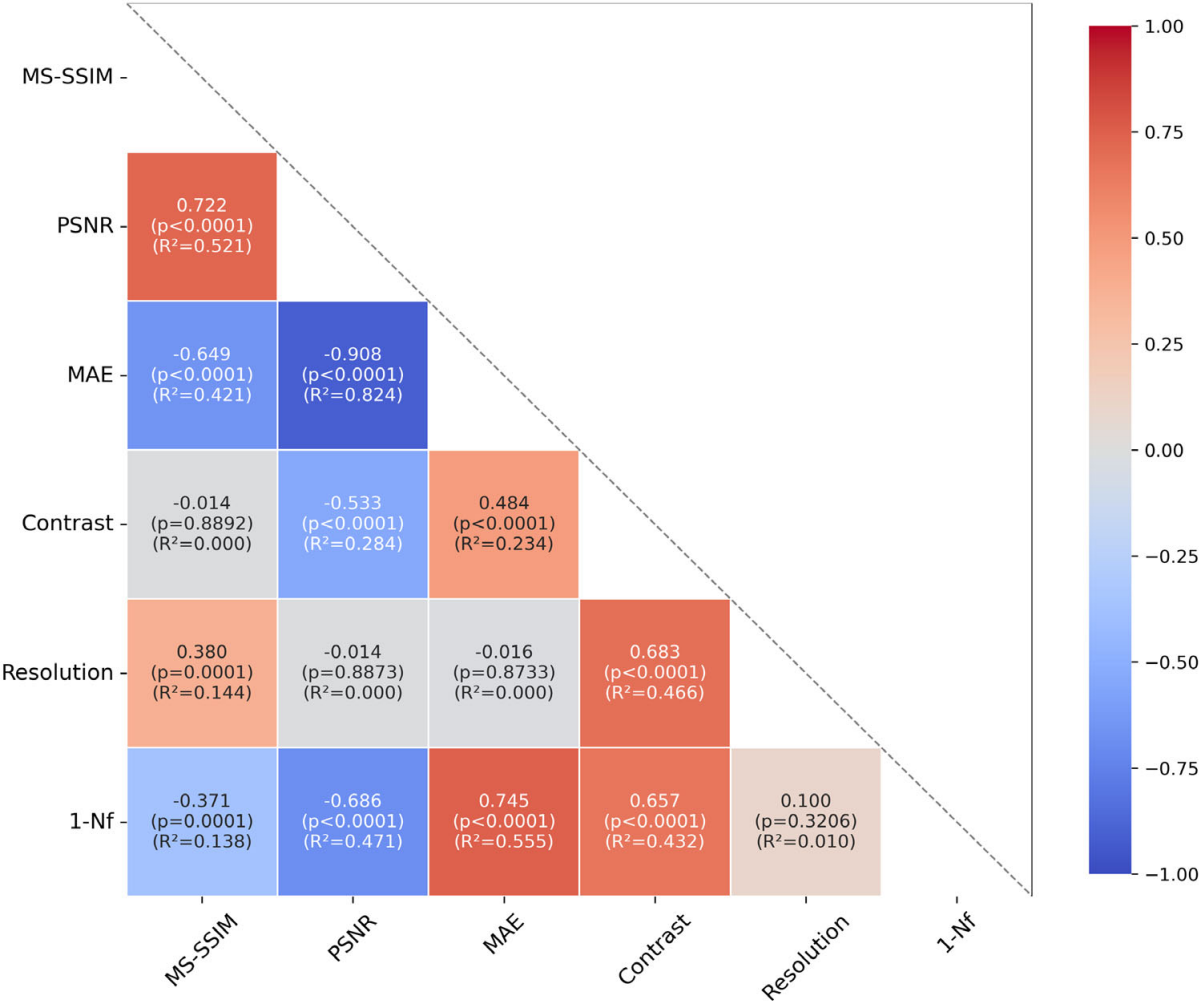
Pairwise Pearson correlation coefficients between all considered image quality metrics across the 100 test images from Figure 2, computed for the CVDM‐generated output images. Correlation is color‐coded (color bar) with color saturation corresponding to correlation strength. The correlation coefficients are given in each box, along with their coefficients of determination (*R* ^2^) and p‐values. The null hypothesis is that the two respective metrics are not linearly correlated. The p‐values are computed using a two‐sided Student's t‐test with *n* − 2 degrees of freedom, where *n* = 100 is the number of observations.

Increasing image sharpness is expected to lower *N*_*f*_, while decreasing image sharpness (blurring) would increase it. A very high *N*_*f*_ value is equally undesirable as a very low one. Since the “target” *N*_*f*_ is 1, whereas the other metrics are monotonic, we correlate the other metrics with the deviation of *N*_*f*_ from 1, 1 − *N*_*f*_. The results are computed on the same set of 100 test images, randomly selected from the BioSR test data set, as outlined in the previous section. **Figure** **3** reports the pairwise Pearson correlation coefficients between all metrics for the CVDM‐generated output images with respect to the corresponding super‐resolution ground‐truth images from the BioSR data set, along with the respective coefficients of determination (*R* ^2^) and p‐values from a two‐sided t‐test.

The null hypothesis for the t‐test is that the two respective metrics are not linearly correlated. The results are computed using SciPy's scipy.stats.pearsonr function^[^ ^46^ ^]^ for Student's t‐distribution with *n* − 2 degrees of freedom, where *n* = 100 is the number of observations. We use a two‐sided test to assess the significance of both positive and negative correlations. We reject the null hypothesis for *p* < 0.05, indicating that the metrics are significantly correlated.

From the data in Figure 3, we observe that MS‐SSIM and PSNR are negatively correlated with 1 − *N*_*f*_, while MAE and Contrast are positively correlated with 1 − *N*_*f*_. All of these correlations have high statistical significance (p‐values ⩽10^−4^). Calculated image Resolution, however, is practically uncorrelated with low statistical significance (p‐value of 0.32). This means that more natural images (1 − *N*_*f*_ closer to 0) on average tend to have better quality (lower MAE, higher MS‐SSIM, higher PSNR). The positive correlation with Contrast is more subtle to interpret. It suggests that images with higher 1 − *N*_*f*_, which are less natural, tend to score higher Contrast. Since there is no “optimal” value for Contrast, however, it could be that those images are in fact less desirable. The average correlation is not informative in this case.

Indeed, most of the average correlations observed have limited predictive power, since the the *R* ^2^ for all statistically significant correlations are below 0.56. This indicates high variability for individual images. Therefore, while the correlations are significant on average, the metrics do not strongly depend on each other for any individual image. The null hypothesis that *N*_*f*_ is redundant with other image metrics can therefore be rejected with high significance. This suggests that *N*_*f*_ can provide useful additional information when evaluating DLSR images.

### 3.3 The Naturalness Factor Reflects Image Quality in a Case Where Other Metrics Fail

Knowing that *N*_*f*_ captures different information than the other image metrics, we proceed to test how it relates to perceived image quality in a scenario where other metrics fail. Pixel‐based metrics typically run into problems when a sharpening or blurring filter is applied to a sparse image consisting mostly of background. In the context of DLSR image generation, we are specifically interested in cases where an artificially blurred image would be classified as “better” by other quality metrics, while *N*_*f*_ would provide correct information.

**Figure 4:**
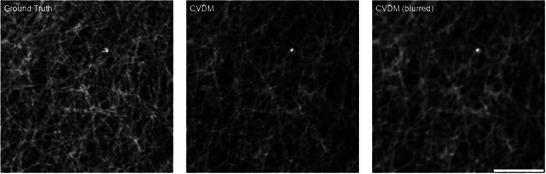
F‐actin images used to test the response of the image quality metrics to blurring (Table 1). From left to right: ground truth super‐resolution image, CVDM‐generated super‐resolution image, and an artificially blurred version (Gaussian blur with kernel size 5 × 5 pixels) of the CVDM‐generated image. The scale bar is 1.5 µm.

We use the trained CVDM to generate a DLSR image of an F‐actin structure. The generated image is then blurred with a Gaussian filter of size 5 × 5 pixels and subsequently re‐normalized to the same intensity range (**Figure** **4**). The image metrics are calculated for the CVDM image and its blurred version using the corresponding super‐resolution ground truth from the BioSR data set as reference image (**Table** **1**). The artificially blurred image achieves better MS‐SSIM, MAE, PSNR, and Contrast with respect to ground truth than the originally generated image. The *N*_*f*_, however, is closer to the target value of 1 for the original CVDM output and worsens for the blurred image, in line with the calculated Resolution metric. This not only correctly provides the information that the original CVDM‐generated image is the better‐resolution SRM image, but the increase in *N*_*f*_ also tells that the second image is blurrier. This suggests that *N*_*f*_ is a useful metric for scoring the visual quality of DLSR images in this example where other metrics fail.

While useful as an illustration of how metrics can fail in a counter‐intuitive way, the present example hinges on re‐normalizing the image after blurring. We show this by repeating the experiment on 500 randomly chosen images of F‐actin structures and 500 images of MT structures. Without intensity range normalization after blurring, the Contrast metric correctly decreases for all tested images. However, when re‐normalizing the intensity range after blurring, 987 out of the 1,000 images show an increased Contrast metric. This is because Gaussian blurring compresses pixel intensities into a narrower range. Normalization then stretches the intensity distribution back to the original range, which increases the variance and thus “improves” the Contrast metric.

### 3.4 The Naturalness Factor is a Useful Prior for CVDM Training

Having established that *N*_*f*_ can capture image quality in cases where other metrics fail, we ask the question whether biasing a generative CVDM toward *N*_*f*_ = 1 improves its performance in DLSR, in the sense that it produces images with better‐resolved small structures.

An image can be transformed into one that has a *N*_*f*_ closer to one by a process called image naturalization.^[^ ^16^ ^]^ This uses histogram equalization to map the gradient distribution of the original image to the prior distribution expected for natural‐scene images, followed by integrating the signal again.^[^ ^7^ ^]^ The thus naturalized image has a *N*_*f*_ closer to one than the original image.

A straightforward way to generate more natural DLSR images therefore is to naturalize the output of CVDM. We call this “CVDM+N” (CVDM plus naturalization). Alternatively, one can train CVDM on pairs of low‐resolution and naturalized super‐resolution BioSR images. We call the resulting model “NCVDM” (naturalized CVDM). Details of the model training are given in the Appendix. We compare these two approaches with the standard CVDM used above, with no naturalization or naturalness prior.

**Figure A1:**
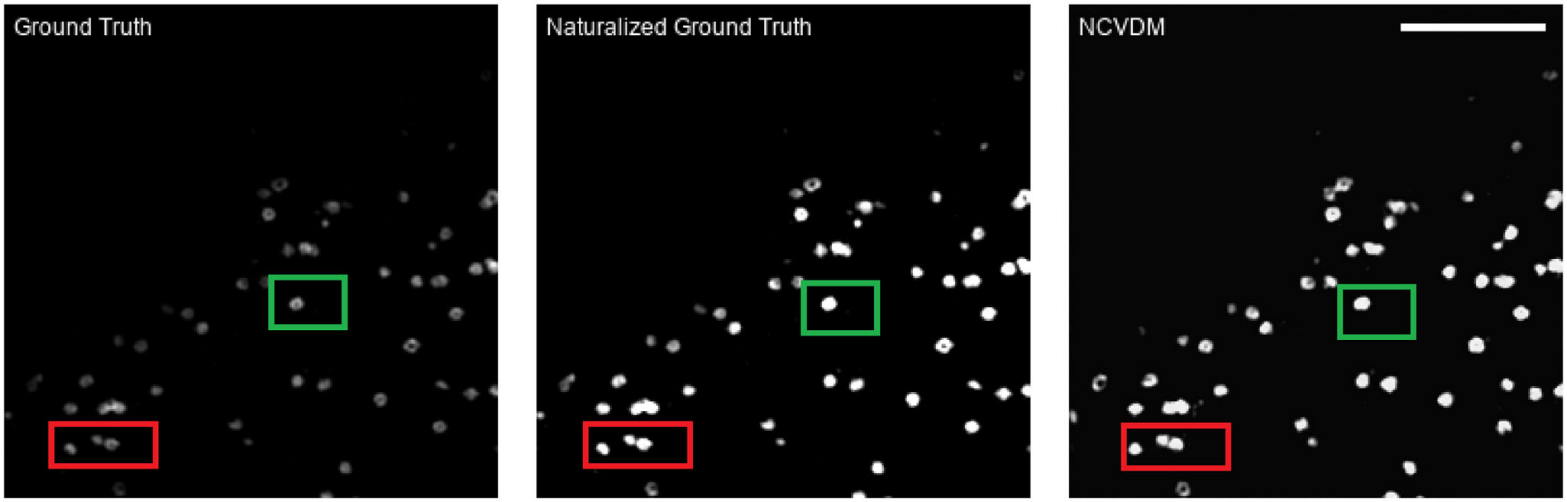
The effect of model misspecification in a case where the naturalness prior is not appropriate. From left to right: ground‐truth image of clathrin‐coated pits (CCPs), naturalized ground truth, and NCVDM output. The colored squares highlight regions where the model misspecification error manifests in a catastrophic loss of super‐resolution structure. The scale bar is 1.5 µm.

We perform this comparison on images of F‐actin and MT. As shown in Figure 2, these filamentous structures in the BioSR data set have ground‐truth images with *N*_*f*_ close to one. A naturalness prior is therefore potentially appropriate for these structures, whereas it is clearly inappropriate for other structures. For example, we do not expect the dot‐like CCP images to benefit from a naturalness prior, as they are far from natural‐scene images in their “salt and pepper” appearance. As shown in Appendix **Figure** **A1**, nevertheless specifying the wrong prior of *N*_*f*_ = 1 in this case leads to distorted reconstructions and a loss of fine details.

**Figure 5:**
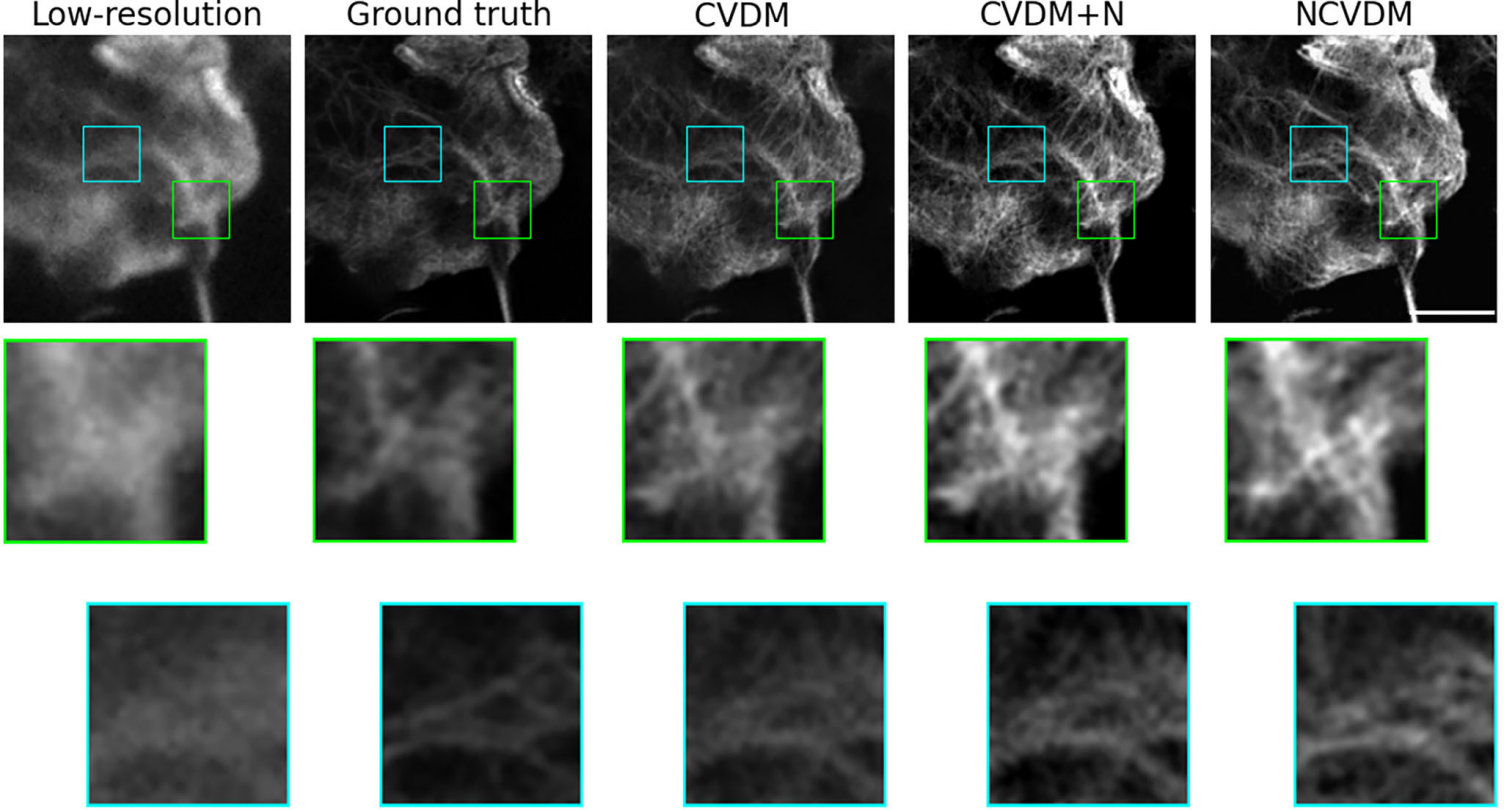
Visual image comparison for an example F‐actin structure. From left to right: low‐resolution input (*N*_*f*_ = 1.989), ground truth (*N*_*f*_ = 2.315), CVDM output (*N*_*f*_ = 1.606), CVDM+N output (*N*_*f*_ = 1.098), and NCVDM output (*N*_*f*_ = 1.103). The scale bar is 1.5 µm. Zoomed insets below each image highlight the detail structures in the respective colored boxes.

**Figure 6:**
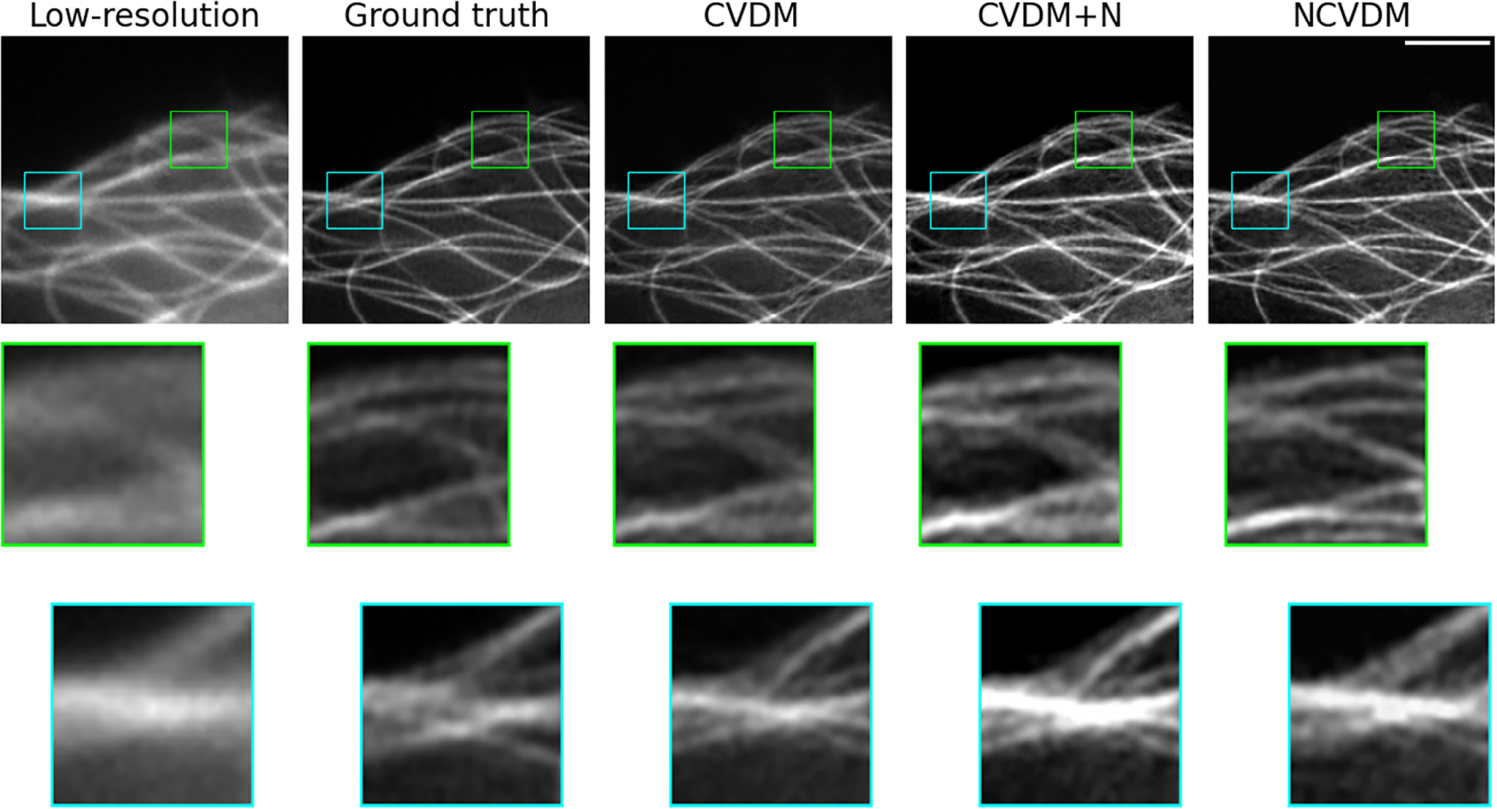
Visual image comparison for an example microtubule structure. From left to right: low‐resolution input (*N*_*f*_ = 1.989), ground truth (*N*_*f*_ = 1.784), CVDM output (*N*_*f*_ = 1.664), CVDM+N output (*N*_*f*_ = 1.112), and NCVDM output (*N*_*f*_ = 1.127). The scale bar is 1.5 µm. Zoomed insets below each image highlight the detail structures in the respective colored boxes.

**Figure 7:**
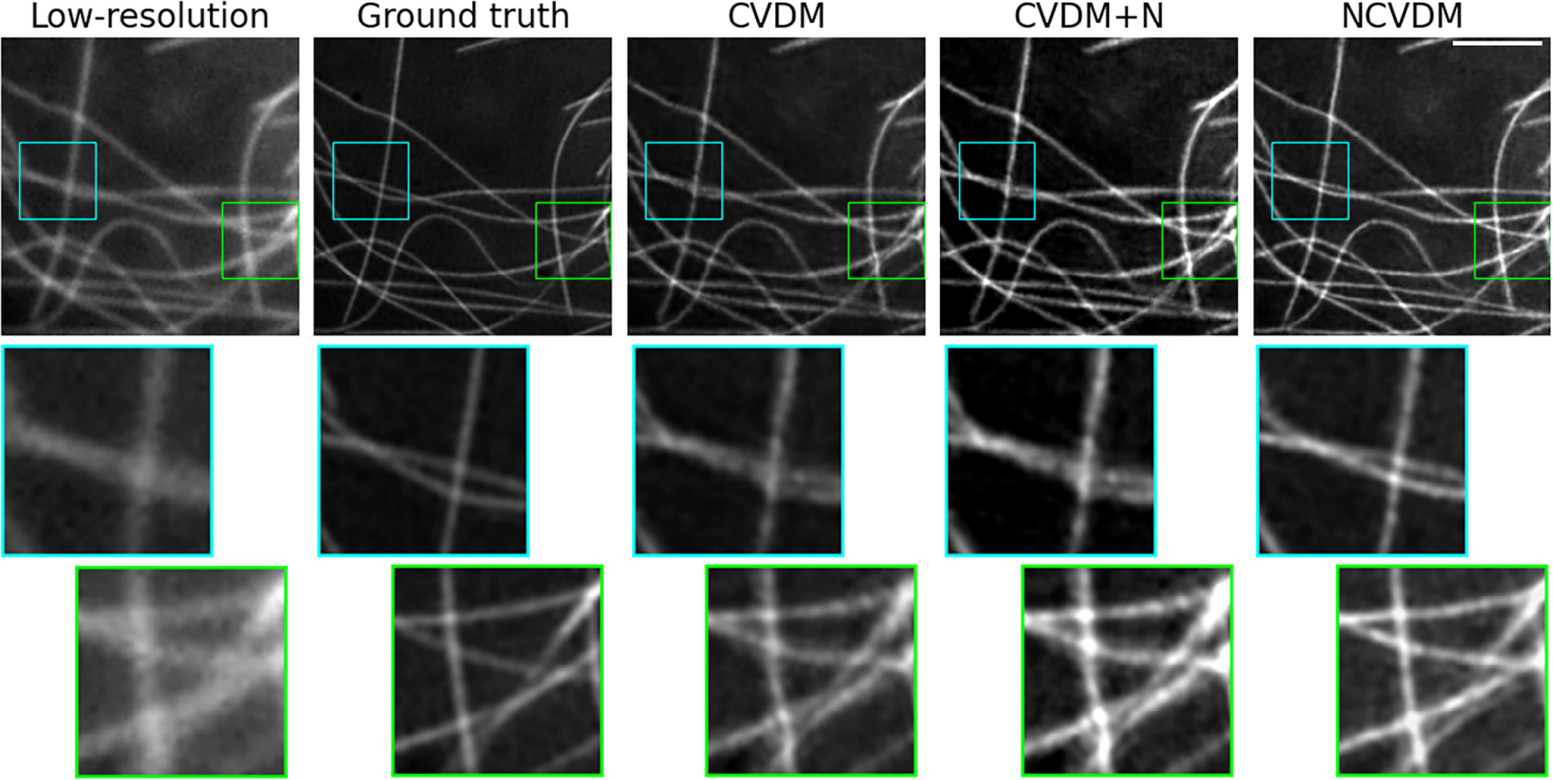
Visual image comparison for an example microtubule structure with close and crossing filaments. From left to right: low‐resolution input (*N*_*f*_ = 1.898), ground truth (*N*_*f*_ = 1.660), CVDM output (*N*_*f*_ = 1.637), CVDM+N output (*N*_*f*_ = 1.036), and NCVDM output (*N*_*f*_ = 1.069). The scale bar is 1.5 µm. Zoomed insets below each image highlight the detail structures in the respective colored boxes.

The image quality metrics of the results for the filamentous structures are summarized in **Table** **2**. As expected, the MS‐SSIM, MAE, and PSNR scores are best for the standard CVDM (numbers in bold). In the Contrast, Resolution, and *N*_*f*_ metrics, CVDM+N and NCVDM perform better, albeit the difference in Resolution is not significant, as already observed by the insignificant correlation in Section 3.2. As expected, the *N*_*f*_ for CVDM+N and NCVDM‐generated images are closer to one in all experiments than those of standard CVDM‐generated images.

Given that *N*_*f*_ is sensitive to image quality (see Section 3.3), this improvement in *N*_*f*_ of the model outputs begs the question whether the resulting images look better to the human eye. Also, is there a visual difference between CVDM+N and NCVDM, while the respective metrics are not significantly different?

**Figures** **5** and **6** show visual comparisons of randomly selected example images of F‐actin and MT, respectively. The outputs of NCVDM and CVDM+N are easier to grasp for the human eye than the standard CVDM output. They appear to have more contrast, as also confirmed by the Contrast metric in Table 2, better brightness range, and more small‐scale details. In this, NCVDM appears to produce images with more natural texture, as is best appreciated in the magnified insets (colored boxes).

**Figure A2:**
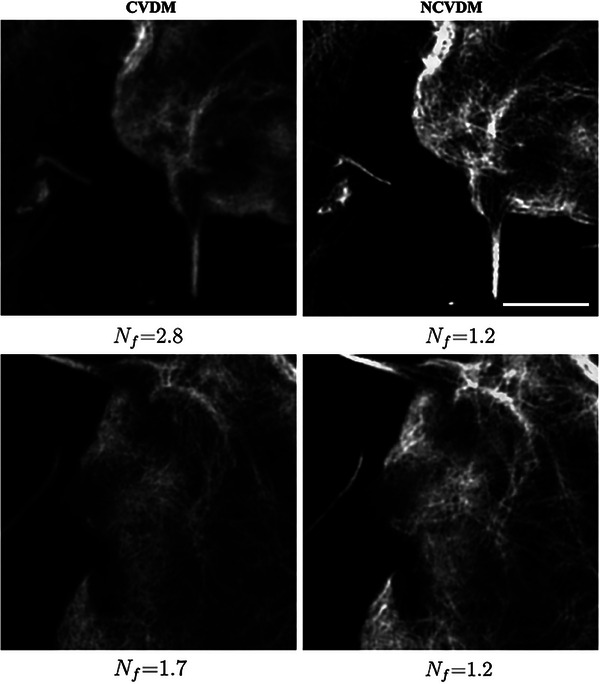
Comparison of CVDM (left column) and NCVDM (right column) outputs for two samples (rows) with dense regions of F‐actin filaments. The scale bar is 1.5 µm.

These observations align with the original CVDM paper^[^ ^24^ ^]^ reporting difficulties in recovering fine‐grained image details due to the spectral bias of neural networks preferring low‐frequency solutions when solving ill‐posed problems.^[^ ^35^, ^36^ ^]^ This causes CVDM to generate more “locally blurry” images, in which adjacent pixels tend to have similar intensities. Post‐hoc naturalization of such a result, while improving contrast and brightness range, will not bring back those details. This effect is most visible where filaments cross or come close to each other. Examples of this are shown in **Figure** **7** with the insets in the boxes magnified below. NCVDM, trained on naturalized images, produces better details in these regions, more closely matching the ground truth. Standard CVDM tends to omit these small‐scale details, while CVDM+N shows reconstruction artifacts, e.g., where filaments cross (large bright “bubbles”). As shown in Appendix **Figure** A2, the benefit of higher image detail in NCVDM also persists in regions of dense filaments where intensity gradients are smaller on average.

Taken together, these examples show that a naturalness prior in generative models of DLSR can reconstruct more sub‐resolution structures and lead to better fine‐scale image detail. This is likely because the naturalness prior generates better‐conditioned gradient distributions for CVDM training, reducing the susceptibility of the model to spectral bias, ultimately learning a generative model that better reproduces high frequencies. This is most noticeable in regions of high filament density, or where filaments cross, as the low‐resolution input image is the least informative there.

We conclude that *N*_*f*_ = 1 can provide a spectral prior in generative CVDM models for DLSR, where it may improve the visual appearance of the generated images. Training a dedicated NCVDM seems to help with spectral bias and yield a generative model with better texture and detail reproduction. Thus, generated super‐resolution images could facilitate early discovery of small biological structures.

## 4 Conclusion and Discussion

We have proposed a spectral prior for deep‐learning super‐resolution (DLSR) fluorescence microscopy image generation. The presented prior regularizes the training images to have gradient and Laplacian statistics more closely matching those of natural‐scene images. This naturalness prior can be computed independently of the training and input data, and it does not require a reference image. Importantly, since natural image statistics universally follow a hyper‐Laplace law,^[^ ^7^, ^8^, ^15^, ^16^ ^]^ the prior can be expressed by a single scalar value, the naturalness factor, which renders it practically useful also as an image quality metric. As we have shown, the naturalness factor as an image quality metric is not redundant with established metrics, providing complementary information about an image's gradient statistics.

Such a gradient‐statistics metric is potentially useful in two settings: First, it can be used to score the output of a DLSR model at inference time, when no ground‐truth is available. Second, it can be used as a prior or bias to improve the performance of generative DLSR models. We have evaluated both use cases.

Using the naturalness factor as an inference score, we have shown that it better captures perceived image quality in cases where other metrics can be misleading, such as when blurring an image. There, *N*_*f*_ is also able to detect whether an image has been blurred. We have also shown that an *N*_*f*_ close to 1 is significantly correlated with image quality as quantified by established metrics.

The correlation between *N*_*f*_ = 1 and average image quality is exploited when using the naturalness factor as a prior or bias in generative DLSR models. We demonstrated this in the state‐of‐the‐art Conditional Variational Diffusion Model (CVDM) for DLSR.^[^ ^24^ ^]^ As a baseline, we trained a CVDM on the BioSR data set containing more than 100,000 matched pairs of diffraction‐limited and super‐resolution images of samples from four biological structures. For the filamentous structures, where the proposed prior is appropriate, we compared the baseline CVDM with a version trained on naturalized training images and with naturalizing the output of the baseline model in a post‐processing step. Image naturalization transforms an image to having a *N*_*f*_ closer to 1 using gradient histogram equalization.^[^ ^16^ ^]^ When naturalization was used as a data regularizer during CVDM training, the model produced images with better *N*_*f*_ and often also with better contrast and Fourier resolution. Nevertheless, improving *N*_*f*_ is not the same as simply improving contrast, since the RMS Contrast metric is insensitive to image structure and assumes pixel independence. Also, while RMS Contrast is a metric, it is not clear what transformation would be the contrast‐analog of naturalization and what the “optimal” contrast value would be in a model prior. The method proposed here is practically computable via the image naturalization transformation,^[^ ^7^, ^16^ ^]^ and *N*_*f*_ has a well‐defined target value of one. This can lead to better microstructure resolution in the generated images, as became apparent when comparing dense image regions with small‐scale structures between NCVDM trained with naturalness prior, and post‐hoc naturalization of the output of standard CVDM.

Of course, the naturalness prior only makes sense in cases where the ground‐truth training data has *N*_*f*_ close to 1. In the BioSR data set, this is the case for filamentous structures (microtubules and F‐actin). Inappropriately using the naturalness prior, for example for images of clathrin‐coated pits, led to a loss of microstructure due to model misspecification errors.

Another limitation of the present work is that we only considered grayscale images. While the naturalness factor has previously been shown to also work on three‐channel RGB color images,^[^ ^16^ ^]^ we are not aware of any multi‐channel SRM data set that could be used to train such models. Also, we used the same target gradient and Laplacian distribution as Gong et al. ^[^ ^16^ ^]^ when defining the naturalness prior. Those reference distributions were determined from natural‐scene images. It might be interesting to explore in the future whether microscopy‐specific invariant reference distributions can be found with respect to which a “microscopy quality factor” could be defined analogously to the naturalness factor. Finally, we fixed the mixing weight θ in the definition of *N*_*f*_ to the value used in previous works. This renders the metric parameter‐free and has been shown to work well for natural‐scene images.^[^ ^7^ ^]^ For a microscopy‐specific version of *N*_*f*_, however, other values of θ might be better, and a parameter screen should be performed. Our goal here was to show that already the standard naturalness prior is promising for DLSR image generation.

Indeed, images generated by naturalization‐regularized NCVDM overall seemed visually more appealing, and small structures were more clearly resolved. Naturalizing the baseline model output improved overall image appearance but did not lead to better sub‐diffraction detail reconstruction. This addresses one of the main reported limitations of DLSR models,^[^ ^24^ ^]^ and we think this is because the model is less prone to spectral bias when trained on images with more natural gradient statistics.

In conclusion, while many open questions and limitations remain, we believe that the naturalness prior contributes interesting new ideas to the field of DLSR microscopy. Its perceptive character, together with its independence from a strong ground‐truth reference, render it complementary to the majority of existing reconstruction priors and image quality metrics. Contributing a more vision‐centric viewpoint on the data, the naturalness factor enables nuanced discussion of model outputs, especially during the exploratory phase of discovery. In addition, our results hint at promising research directions using the naturalness factor as a prior or bias when training DLSR models. This could lead to better‐conditioned input gradient statistics, reducing the spectral bias of the models and generating more fine‐textured output. While these observations are promising, they require further investigation and comparison with other types of bias, or with a *N*_*f*_‐term in the loss (which is not straightforward for a diffusion model). In all of this, however, meaningful application of the naturalness factor as a model prior remains limited to images of structures that are supposed to appear natural in the first place. Nevertheless, since the availability of annotated high‐resolution ground truth is a key limiting factor when using deep learning in microscopy, the general concept behind the reference‐free naturalness prior could contribute to further advancing the field.

## Conflict of Interest

The authors declare no conflict of interest.

## Data Availability

The data that support the findings of this study are openly available in GitLab at https://gitlab.mn.tu‐dresden.de/the‐naturalness‐factor‐in‐generative‐deep‐learning‐models‐of‐super‐resolution‐microscopy/.

## CVDM and NCVDM Implementation and Training Details

We used the publicly available implementation of CVDM^[^ ^47^ ^]^ with the same architecture and configuration as in previous work.^[^ ^24^ ^]^ CVDM and NCVDM were independently trained on the same number of pairs of low‐resolution and (naturalized for NCVDM) super‐resolution BioSR images using the same training parameters.

The training data consisted of 101,880 image pairs, obtained from ≈92% of the BioSR image pairs by data augmentation (cropping to size 256 × 256, flipping, rotating).^[^ ^24^ ^]^ The remaining ≈8% of the BioSR image pairs were set aside for model evaluation, generating a test set of 8,544 matched image pairs after data augmentation, 2,136 for each of the four biological structures.

Each model was trained for 500,000 iterations with batch size two, generation time step *T* = 200, and a learning rate of 0.0001. For NCVDM, the super‐resolution training images were naturalized prior to training using the publicly available implementation of image naturalization in the MOSAICsuite plugin for Fiji/ImageJ.^[^ ^48^ ^]^ Training took 50 h for each model on a workstation equipped with a Nvidia RTX 3090 GPU, with 24 GB of GPU memory, and a dual‐socket Intel Xeon CPU with 20 physical cores and 40 threads.

For inference, the generation time step was set to *T* = 500, and the batch size was four. CVDM evaluation was performed on the 8,544 test image pairs, 2,136 for each biological structure, and every generated image was saved. This led to a total inference time of 86 h for all test images on the same hardware as used for training.

Due to compute resource constraints, NCVDM inference was performed on 100 randomly selected image pairs from the test data set, 25 for each biological structure. These images are available in our GitLab repository at: https://tud.link/68rs4e.

To ensure a class‐balanced comparison, the same 100 test images were also selected from the 8,544 CVDM output images when comparing NCVDM with CVDM. For CVDM+N, the same 100 CVDM‐generated images were additionally naturalized in a post‐processing step using the MOSAICsuite plugin for Fiji/ImageJ.^[^ ^48^ ^]^

## Validation of the Present CVDM Implementation and Training

Table A1 compares CVDM output metrics on the 8,544 test data set between the present implementation and the published reference implementation.^[^ ^24^ ^]^ The results are comparable given the stochastic nature of the diffusion process, indicating that our implementation works correctly and that we have used the model correctly.

## Demonstration of Model Misspecification Error for CCP Images

Clathrin‐coated pits (CCPs) are punctate structures whose images have ground‐truth *N*_*f*_ much larger than one (cf. Figure 2). As shown in Figure A1, nevertheless, imposing the prior *N*_*f*_ = 1 in this case, where it is not appropriate, leads to distorted model output. This is especially seen in the examples highlighted by the colored boxes, where the hollow CCP structures are wrongly filled in, both in the naturalized training data and in the NCVDM output. This artifact defeats the purpose of SRM, clearly showing the detrimental effect of model misspecification.

## NCVDM Performance Does not Depend on Image Sparsity

The quality of NCVDM results does not depend on image sparsity. This is shown in the example in Figure A2, exhibiting large areas with dense F‐actin filaments and almost flat intensity distribution. Also in those areas, NCVDM produces visually better results than CVDM. This is because the naturalness prior does not simply act on the gradient values, but it is a spectral prior over the entire gradient and Laplacian distributions.
